# Production of Activated Carbon from Grape Pomace (*Vitis labrusca*) by Chemical Activation with ZnCl_2_ and Its Application in Phenol Adsorption

**DOI:** 10.1021/acsomega.5c08993

**Published:** 2026-01-28

**Authors:** Amanda Ferreira Scholant, Márcia Moreira, Alice Neri da Silva Sousa, Keli Arruda da Silva, Nauro da Silveira, Débora Pez Jaeschke, Luiz Antonio de Almeida Pinto, Tito Roberto Sant’ Anna Cadaval

**Affiliations:** School of Chemistry and Food, 67820 Federal University of Rio Grande—FURG, Italia Avenue 8 km, 96203-900 Rio Grande, Rio Grande do Sul, Brazil

## Abstract

This study investigated the production of activated carbon from *Vitis labrusca* grape pomace through chemical activation with ZnCl_2_ and subsequent carbonization at varying temperatures and times using a fractional factorial design. The best performance for phenol adsorption was obtained at 800 °C for 120 min, achieving a maximum adsorption capacity of 54.04 mg g^–1^. The activated carbon presented a high specific surface area (1017.58 m^2^ g^–1^), meso- and micro-porous structure (type I and IVa isotherms with H4 hysteresis), oxygenated functional groups, and thermal stability. Adsorption kinetics were studied at different phenol concentrations (50–150 mg L^–1^), and the experimental data were fitted to pseudo-first-order, pseudo-second-order, and Elovich models. The results revealed that the pseudo-second-order model better described the kinetic data. Regarding the equilibrium studies, the maximum adsorption capacity was 180 mg g^–1^. Isotherm modeling indicated that the Freundlich model was the most appropriate. Thermodynamic analysis indicated that the adsorption process is spontaneous and favorable, with Δ*G*° values of −18.85, −20.03, and −21.53 kJ mol^–1^ at 25, 45, and 55 °C, respectively, Δ*H*° of 6.2 kJ mol^–1^, and Δ*S*° of 83.8 J mol^–1^ K^–1^ at 45 °C, demonstrating that higher temperatures enhance adsorption. Reuse tests showed that after five adsorption–desorption cycles, the adsorbent maintained its mechanical integrity and physical stability. These findings confirm that grape-pomace-derived activated carbon is a sustainable and effective material for phenol removal from aqueous solutions.

## Graphical abstract

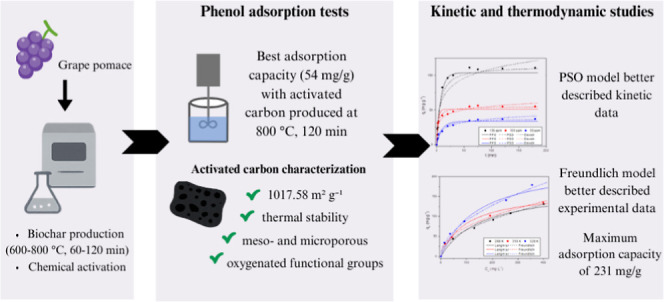

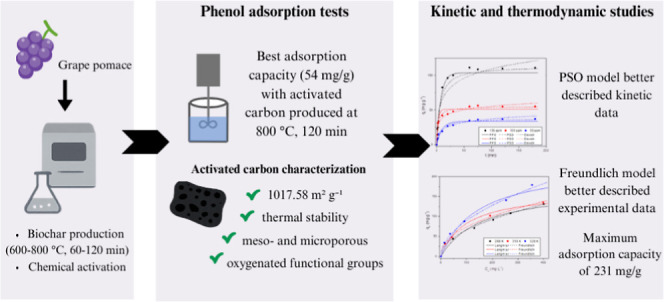

## 1 Introduction

Phenol is recognized as a highly toxic and environmentally hazardous compound. It is listed as a priority pollutant by regulatory agencies due to its toxicity, carcinogenic and teratogenic effects, and potential to cause harm even at low concentrations. According to international standards, the maximum allowable concentration of phenol in drinking water is 0.001 mg L^–1^, as recommended by the World Health Organization (WHO).^1^ The United States Environmental Protection Agency (EPA) sets a limit of 2 mg L^–1^ for drinking water.^2^ In Brazil, CONAMA Resolution No. 397/2008 establishes a discharge limit of 0.5 mg L^–1^ for total phenols in industrial effluents, serving as the main national reference.^3^

Phenol is widely used in various industrial sectors, including papermaking, petrochemical processing, oil refining, and coal coking. The discharge of phenol-contaminated wastewater from these activities poses serious risks to aquatic ecosystems and human health, necessitating effective treatment before environmental release.^4^ Although various physical, chemical, and biological methods have been developed for phenol removal, adsorption remains one of the most widely used approaches due to its low capital cost, operational simplicity, and versatility in targeting a broad range of contaminants.^5,6^

In this context, activated carbon is widely used across various industrial sectors due to their high adsorptive capacity, which stems from its large surface area and highly porous structure, making it particularly effective in water and air purification processes, as well as in the removal of contaminants from industrial effluents.^7,8^ To obtain activated carbon, the carbonized material undergoes physical, chemical, or biological activation processes to improve its properties, such as specific surface area and micropore volume.^9,10^ Chemical methods, using acids, alkalis, or impregnation modify the functional groups of the material surface and improve adsorption capacity and selectivity.^11^ Particularly, ZnCl_2_ promotes the degradation of biopolymers and removes oxygen-containing groups. When melted (above 263 °C), it penetrates and swells the biomass structure, facilitating pore development. It also forms intermediate compounds such as Zn_2_OCl_2_·2H_2_O, which decompose at higher temperatures to release gases that enhance microporosity. Above 600 °C, embedded ZnO reacts with carbon to produce gases that further expand the porous network. Volatilization of ZnCl_2_ above its boiling point (732 °C) also contributes to pore generation.^12^

The resulting structure of activated carbon is characterized by a system of interconnected pores, which are classified as micropores, mesopores, and macropores.^11^ Characterizing this pore structure is crucial for assessing the material selectivity in adsorbing a wide range of substances. Furthermore, understanding the physicochemical interactions between activated carbon and the adsorbed compounds is essential for evaluating its performance and cost-effectiveness across various industrial and environmental applications.^13,14^

Among the precursor materials used for activated carbon production, agricultural residues, wood, nut, and fruit shells, and synthetic polymers are commonly utilized.^15,16^ Grapes are among the most widely cultivated fruits worldwide, with a third of the total production used in winemaking.^17,18^ In 2024, 29.4 million tons of grapes were used for wine production. These residues, depending on the winemaking process, can generate between 10 and 30% of waste as a mass of crushed grapes.^19^ Vine pruning residues, exhausted grape marc, and vinasse are byproducts of the winery and distillery industries. These wastes hold significant potential for valorization, including applications in composting, biochar production, polyphenol, and tannin extraction, as well as use as adsorbents.^20^

Activated carbon from grape residues has already been used to adsorb contaminants, such as metals (Ag­(I), Cr­(VI), Pb­(II)),^21,22^ dyes^23^ and medicines.^24^ However, no studies have investigated its application for phenol removal. This work addresses this gap by producing activated carbon from grape pomace under different pyrolysis conditions and evaluating its structural properties. Adsorption performance was analyzed through isotherms, kinetics, and thermodynamics, providing insight into the process mechanisms. In addition, the reusability of the material was assessed, highlighting its potential for sustainable wastewater treatment.

## 2 Materials and Methods

### 2.1 Activated Carbon Production

The grape pomace (*Vitis labrusca*, Bordô variety) was supplied by the company TANAC (Montenegro, RS, Brazil). The production of activated carbon followed a methodology comprising dethawing, pretreatment, impregnation, carbonization, washing, and drying steps. For the pretreatment, approximately 600 g of dethawed grape pomace was dried at 70 °C to 10% of moisture content. The dried material was ground using a knife mill (Willey, no. 3, USA) equipped with a 1 mm mesh. Subsequently, the resulting powder was sieved (Tyler series), and the fraction that passed through the 20-mesh sieve (850 μm) and was retained on the 48-mesh sieve (300 μm) was collected for further use.

After pretreatment, the materials were impregnated with ZnCl_2_ and then carbonized. During impregnation, the ground biomass was soaked in 200 mL of a ZnCl_2_ solution in a 1:1 ratio (activating agent to biomass) and continuously stirred at room temperature for 24 h. Afterward, the material was filtered and dried in an oven at 110 °C for 24 h. Carbonization was carried out in a muffle furnace (Q.318.24, Quimis) using porcelain capsules at 600 °C, 700 °C, and 800 °C for 60, 90, and 120 min, with a heating ramp of 19 ± 4 °C min^–1^, following the factorial experimental design presented in Table 1.

**Table 1:** Coded and Experimental Values of the 2^2^ Factorial Design Performed to Evaluate the Effect of Temperature and Time on the Carbonization of Grape Pomace

independent variables	–1	0	+1
temperature (°C)	600	700	800
time (min)	60	90	120

Afterward, the activated carbons were treated with 250 mL of a 0.5 mol L^–1^ HCl solution to remove previously impregnated Zn­(II). The washing process was conducted under reflux at 95 °C with continuous stirring for 30 min. Then, the activated carbons were rinsed with distilled water until the pH reached neutrality and subsequently dried in an oven at 110 °C for 24 h.^25^ The produced activated carbons were evaluated regarding their performances in adsorption tests, as explained in Section 2.3.1.

### 2.2 Characterization of the Activated Carbon

#### 2.2.1 Nitrogen Adsorption–Desorption Isotherms and Particle Mean Diameter

The specific surface area (*A*_s_), average pore diameter, and total pore volume were evaluated through nitrogen adsorption–desorption isotherms at 77 K using an automated gas sorption system (Nova 4200e, Quantachrome Instruments, USA). Before measurements, the samples were degassed at 573 K for 12 h. The Brunauer–Emmett–Teller (BET) method was applied to determine the specific surface area, while the Barrett–Joyner–Halenda (BJH) method was used to assess pore size distribution. The mean particle diameter (*D*_p_, μm) was measured by sieving using sieves 48 to 150 from the Tyler series.

#### 2.2.2 Scanning Electron Microscopy

Scanning electron microscopy (SEM) was employed to characterize the samples by using a JEOL JSM-6060 microscope (Japan). The samples for SEM analysis were previously deposited on a carbon tape and prepared by sputter-coating the surfaces with Au.

#### 2.2.3 Fourier Transform Infrared Spectroscopy (FTIR)

FTIR (PRESTIGE 21, Shimadzu) analyses were performed to identify the functional groups on the sample surfaces. The diffuse reflectance technique was used, in which the samples were ground with potassium bromide (KBr), and the spectra were recorded at room temperature, in the range of 4000–600 cm^–1^, with 45 scans and a resolution of 4 cm^–1^, in the ratio of 1:6.

#### 2.2.4 Thermogravimetric Analysis (TGA/DTG)

Thermal analyses of the activated carbon samples were performed using a thermogravimetric analyzer (TGA-50, Shimadzu, Japan). Samples were also placed in sealed aluminum pans with an empty pan as a reference. Measurements were conducted under a nitrogen atmosphere (50 mL min^–1^ flow rate) from 25 to 800 °C at a heating rate of 10 °C min^–1^. Meanwhile, after each TG experiment, the first derivative of the obtained TG profile, i.e., derivative thermogravimetric analysis (DTG), was applied to evaluate the weight loss rate.

#### 2.2.5 Differential Scanning Calorimetry

Thermal analyses of the activated carbon samples were performed using a differential scanning calorimeter (DSC-60, Shimadzu, Japan). Samples were also placed in sealed aluminum pans with an empty pan as reference. Measurements were conducted under a nitrogen atmosphere (50 mL min^–1^ flow rate) from 25 to 300 °C, at the same heating rate of 10 °C min^–1^. To calculate the enthalpy (Δ*H*), TA-60WS software was used, selecting the initial and final base of the transition peak.

### 2.3 Adsorption Experiments

#### 2.3.1 Activated Carbon Performance

Phenol adsorption tests were conducted by using the activated carbons prepared under the conditions described in Section 2.1. The initial phenol solution concentration in water was fixed at 100 mg L^–1^ (pH 6.5), the adsorbent dosage was 1 g L^–1^, and stirring was performed at 150 rpm using an orbital shaker (Nova Ética, model 109-1, Brazil). After the tests, the solutions were filtered using filter paper and the phenol concentration in the aqueous phase was determined by UV–vis spectroscopy at 270 nm (Shimadzu, UV240, Japan).

#### 2.3.2 Adsorption Kinetic Experiments

Kinetic adsorption curves were obtained using activated carbon synthesized under the optimal conditions identified through the fractional factorial design. The initial adsorbent dosage was set at 1 g L^–1^, with agitation at 200 rpm. Phenol concentrations of 50, 100, and 150 mg L^–1^ were investigated (pH 6.5). Samples were taken at predetermined time intervals (5–180 min) and filtered using filter paper. The phenol concentration in the aqueous phase was determined by spectroscopy at 270 nm. All experiments were performed in triplicate. The adsorption capacity at time *t* (*q*_*t*_) was calculated using eq 1.^26^

1 $$q_{t}=\left(\frac{C_{0}-C_{t}}{m}\right)V$$

in which *C*_0_ and *C*_*t*_ are the initial and equilibrium concentrations of the adsorbate, respectively (mg L^–1^), *m* is the mass of the adsorbent (g), and *V* is the volume of the solution (L). Experiment data were fitted to pseudo-first order, pseudo-second order, and Elovich models, which are presented in eqs 2–4, respectively.

2 $$q_{t}=q_{1}\left[1-\exp\left(-k_{1}t\right)\right]$$

3 $$q_{t}=\frac{t}{\left(1/k_{2}q_{2}^{2}\right)+\left(t/q_{2}\right)}$$

4 $$q_{t}=\frac{1}{a}\;\ln\left(1+abt\right)$$

where *q*_*t*_ is the adsorption capacity at time *t* (mg g^–1^), *k*_1_ and *k*_2_ are the rate constants of pseudo-first (min^–1^) and pseudo-second-order (g mg^–1^ min^–1^) models, respectively, *q*_1_ and *q*_2_ are the theoretical values for the adsorption capacity (mg g^–1^), *a* is the initial sorption rate due to d*q*/d*t* with *q*_*t*_ = 0 (mg g^–1^ min^–1^), and *b* is the desorption constant of the Elovich model (g mg^–1^).

#### 2.3.3 Adsorption Thermodynamics

Adsorption isotherms were evaluated at 25, 45, and 55 °C, with initial phenol concentrations ranging from 50 to 400 mg L^–1^ (pH 6.5). Each solution was prepared to a final volume of 100 mL and agitated at 100 rpm in a thermostatic shaker (Fanem 315 SE, Brazil) for 24 h. Afterward, the suspensions were filtered and the residual dye concentration in the filtrate was quantified. The phenol concentration in the aqueous phase was determined by spectroscopy at 270 nm. The adsorption capacity at equilibrium (*q*_e_) was determined by eq 5.

5 $$q_{e}=\frac{V\left(C_{0}-C_{e}\right)}{m}$$

in which *C*_0_ is the initial dye concentration in the liquid phase (mg L^–1^), *C*_e_ is the dye equilibrium concentration in liquid phase (mg L^–1^), *m* is amount of adsorbent (g), and *V* is the volume of solution (L).

The equilibrium isotherms of phenol adsorption were fitted using Langmuir and Freundlich isotherms models. The phenol adsorption was also studied by estimation of the thermodynamic parameters, Gibbs free energy change (Δ*G*, kJ mol^–1^), enthalpy change (Δ*H*, kJ mol^–1^), and entropy change (Δ*S*, kJ mol^–1^ K^–1^).^27,28^

### 2.4 Desorption and Reuse Assays

Desorption and reuse studies were adapted from Lessa et al.^29^ and conducted with phenol solutions at 100 mg L^–1^. For the reuse assays, 0.05 g of the adsorbent was added to 100 mL of a phenol solution, and desorption was performed in 100 mL of NaOH solution (1 mol L^–1^). Both the adsorption and desorption assays were kept under constant agitation at 25 °C for 1 h. After each cycle, the samples were dried at 105 °C for 12 h, and the activated carbon was reused for phenol adsorption under the same conditions.

The desorption removal rate (*R*, %) was calculated as follows (eq 6)^30^

6 $$R\;\left(\%\right)=\frac{\left(C_{0}-C_{e}\right)}{C_{0}}\times 100$$

in which *C*_0_ is the initial drug concentration (mg L^–1^) and *C*_e_ is the drug concentration after adsorption equilibrium (mg L^–1^).

### 2.5 Statistical and Regression Analysis

The experimental data from the full 2^2^ factorial design were analyzed using a polynomial regression model, presented in eq 7.

7 $$Y=\beta_{0}+\beta_{1}\times X_{1}+\beta_{2}\times X_{2}+\beta_{12}\times X_{1}\times X_{2}$$

in which *Y* represents the response variable, *X*_1_ and *X*_2_ are the independent variables (factors), and β represents the regression coefficients. The central point of the experimental design was performed in triplicate.

Adsorption kinetics and thermodynamics experiments were conducted in triplicate. The parameters were estimated by fitting the models to the experimental data using nonlinear regression with the quasi-Newton estimation method. The quality of the fit was evaluated using the coefficient of determination (*R* ^2^) and the average relative error (ARE). The calculations of model parameters and statistical analysis were performed using Statistica 7.0 software (Statsoft, USA).

## 3 Results and Discussion

### 3.1 Evaluation of Activated Carbon Performance in Adsorption Experiments

Table 2 presents the results for the phenol adsorption capacity at equilibrium. The maximum adsorption capacity (54.04 mg g^–1^) was obtained at 800 °C for 120 min, and the minimum adsorption capacity (40.42 mg g^–1^) was obtained at 600 °C and 60 min. The effects and regression coefficients of the polynomial equation are presented in Table 3. The results showed that the temperature of carbonization (linear and quadratic effects) significantly influenced phenol adsorption capacity. Temperature exhibited a positive effect, which can be attributed to the enhancement of surface area and porosity in the activated carbon at higher values. Although time exhibited a *p*-value slightly above the conventional threshold (*p* = 0.09), it was kept in the model due to its practical relevance and known influence on carbon structure development. Time of carbonization plays a key role in volatile release and pore formation, which are directly related to adsorption capacity. Furthermore, it has been found that longer residence time allows greater heat diffusion through the particles, leading to increased pore formation and, consequently, a larger surface area.^31^ Fadhil and Kareem^32^ explained that longer pyrolysis time and temperature result in a larger surface area due to additional removal of volatile compounds, resulting in new micropores.

**Table 2:** Phenol Adsorption Capacity Responses at Equilibrium according to the Matrix of the Full Factorial Design Matrix^a^

experiment	temperature (°C)	time (min)	*q*_e_ (mg g^–1^)
1	600	60	40.42 ± 0.54
2	800	60	53.11 ± 1.02
3	600	120	42.73 ± 1.43
4	800	120	54.04 ± 0.77
5	700	90	51.51 ± 0.55
6	700	90	52.66 ± 0.83
7	700	90	49.62 ± 0.02

a Mean values followed by standard deviation of the mean.

**Table 3:** Effects, Regression Coefficients, and *p*-Values of the Parameters of the Models Obtained for Phenol Adsorption Capacity (*q*_e_, mg g^–1^)

parameters^a^	effect	coefficient^b^	*p*-value
mean	48.80	48.80	<0.05
temperature (°C) (L)	11.99	5.99	<0.05
temperature (°C) (Q)	3.69	1.84	<0.05
time (min) (L)	1.62	0.81	0.09
temperature^a^ time	–0.69	-	0.43
*R* ^2^		0.9758	
*F*_calculated_		84.04	
*F*_tabulated_		4.34	

a L is linear effect and Q is quadratic effect.

b Coefficients were obtained for the reduced model.

The literature reports similar behavior in the production of biochar and activated carbon from different agro-industrial biomasses. For instance, for hazelnut shell biochar there was a significant increase in adsorption capacity with increasing temperature, while residence time, despite having a positive impact, was not significant.^31^ Moreover, Suresh Babu et al.^33^ found that, in the production of biochar from coconut shells and mixed wood residues, there was a significant change in surface area as residence time increased, which directly affects adsorption.

Figure 1 presents the response surface for the phenol adsorption capacity. As observed, higher capacities were achieved at elevated pyrolysis temperatures and longer residence times. Accordingly, activated carbon produced at 800 °C for 120 min was selected, as it exhibited the highest equilibrium adsorption capacity for phenol. This material was subsequently characterized and employed in kinetic and equilibrium isotherms studies.

**Figure 1:**
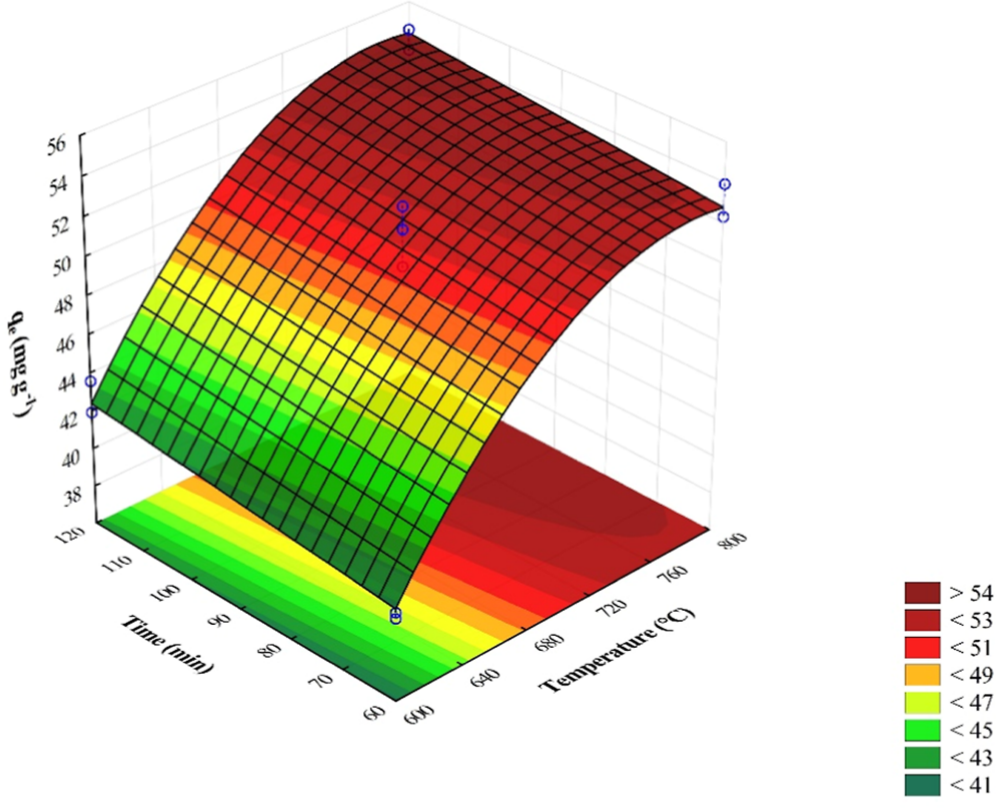
Effect of pyrolysis temperature and time on the phenol adsorption capacity of activated carbon.

### 3.2 Characterization of the Activated Carbon

The physicochemical characteristics of the activated carbon produced under the most suitable condition from the factorial design (800 °C, 120 min) are presented as follows.

#### 3.2.1 Nitrogen Adsorption–Desorption Isotherms and Particle Mean Diameter

Figure 2 shows the nitrogen adsorption–desorption isotherms and pore size distribution of the activated carbon. The curves correspond to a combination of type I and type IVa isotherms with an H4 hysteresis loop, indicating a micro–mesoporous structure.^34^ This behavior is consistent with the literature for ZnCl_2_-activated carbons obtained at high temperatures, which promote pore widening and mesopore formation. The formation of micro- and mesopores is mainly associated with temperatures above 750 °C, at which severe degradation of activated carbon into noncondensable gases occurs, leading to pore widening.^35,36^ In addition, chemical activation with ZnCl_2_ promotes pore development by preventing tar deposition on the carbon surface and enhancing its decomposition.^32^ Da Silva et al.^37^ produced biochar from grape bagasse (700, 800 and 900 °C), and the chars obtained at 800 and 900 °C were classified as type IV with H4 hysteresis. Biochar from various agro-industrial residues within 400 to 700 °C range commonly present H4 hysteresis and type I and IV isotherms.^38^

**Figure 2:**
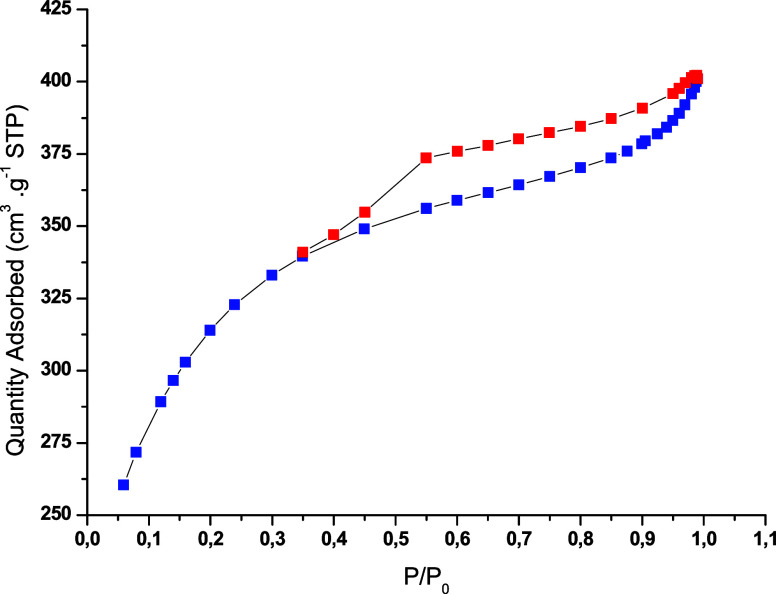
Nitrogen adsorption–desorption isotherms for activated carbon obtained from grape pomace.

Table 4 presents the specific surface area (*A*_s_), total pore volume, average pore size, and average particle diameter (*D*_p_). The results indicate that the material exhibits favorable characteristics for adsorption applications, with a predominance of mesopores, as previously discussed. Regarding the specific surface area, the obtained value was considered high, in comparison with other studies. These results were possibly achieved due to the chemical activation process. For instance, Da Silva et al.^37^ reported much lower surface areas (0.69–20.82 m^2^ g^–1^) for biochars from grape pomace without activation. Demiral and Güngör^39^ obtained surface areas ranging from 292 to 1455 m^2^ g^–1^ for activated carbons produced from grape pomace using H_3_PO_4_, while other studies have also demonstrated high surface areas for ZnCl_2_-activated materials. For example, *Syzygium cumini* leaf-derived activated carbon exhibited a surface area of 1340.66 m^2^ g^–1^.^40^ In contrast, activated carbon obtained from peach stones showed a considerably lower surface area (approximately 50 m^2^ g^–1^), indicating that both the precursor type and activation method strongly influence the textural properties of the final material.^41^ For comparison, commercial activated carbon generally present surface areas ranging from 900 to 1000 m^2^ g^–1^.^42,43^

**Table 4:** Properties of the Activated Carbon Obtained under the Most Suitable Condition of the Fractional Factorial Design

property	
specific surface area (m^2^ g^–1^)	1017.58
total pore volume (cm^3^ g^–1^)	0.620
average pore size (Å)	24.382
*D*_p_ (μm)	203.0

#### 3.2.2 Scanning Electron Microscopy

Figure 3 shows SEM images obtained at 400× (Figure 3a) and 2000× (Figure 3b) magnifications. The images revealed a highly porous surface, which is a fundamental characteristic of activated carbon. The images corroborate the results obtained for the analysis of nitrogen adsorption–desorption isotherms; a heterogeneous distribution of pores is observed, indicating the presence of micro- and mesopores in the material.

**Figure 3:**
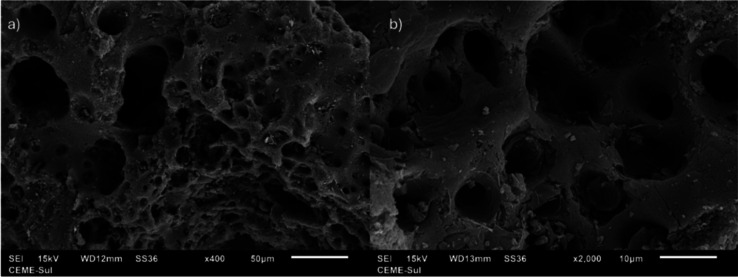
SEM images from activated carbon from grape pomace at different magnifications: (a) ×400; (b) ×2000.

#### 3.2.3 Fourier Transform Infrared Spectroscopy (FTIR)

Figure 4 shows the FTIR spectrum obtained for grape pomace activated carbon before and after adsorption. The spectrum of the activated carbon before adsorption revealed oxygen-containing functional groups (−OH, C=O, and C–O) that are capable of interacting with phenol molecules via hydrogen bonding and π–π interactions. The band observed around 3400 cm^–1^ is an indication of the presence of hydroxyl groups (−OH) of alcohol, carboxylic acid, or phenol groups. The band observed around 3000–2800 cm^–1^ corresponds to symmetric and asymmetric stretching vibrations on aliphatic −CH_2_. The band observed around 2300 cm^–1^ is the ester group characteristic peak.^44^

**Figure 4:**
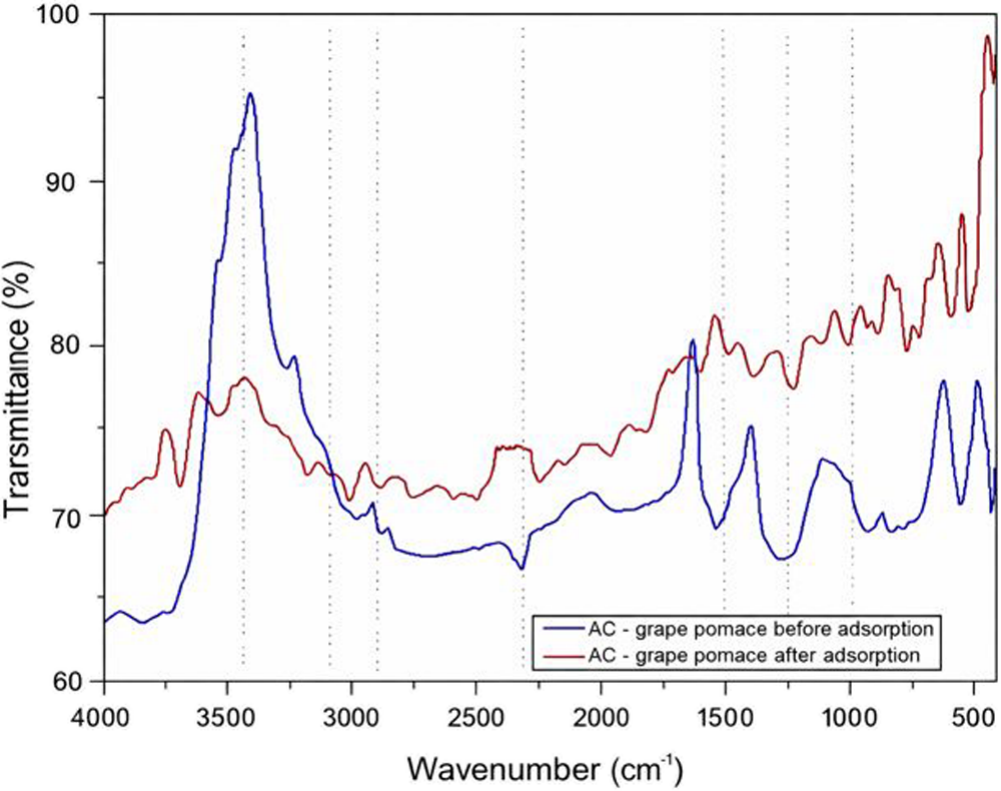
FTIR of the activated carbon from grape pomace before and after adsorption.

The presence of C=C bonds is confirmed by an absorption band near 1600 cm^–1^, reflecting the graphitic or aromatic nature of the carbon. The signal around 1400 cm^–1^ can be linked to C–N stretching in amides or amines, as well as possible C–H bond vibrations. Finally, the band observed around 1000 cm^–1^ is attributed to C–O stretching modes from functional groups such as carboxyl, alcohol, or phenol.^37,45^ The presence of bands indicating oxygen-containing functional groups confirms the chemical activation of carbon.^46^ The spectrum is consistent with activated carbons of vegetal or mineral origin, which typically have a moderately oxidized surface.^32,33^ The reduction in the overall band intensity after adsorption suggests partial coverage of the surface-active sites by adsorbed molecules.

#### 3.2.4 Thermogravimetric Analysis (TGA/DTG)

Figure 5 presents the thermogravimetric analysis (TGA) curve and its derivative (DTGA) of activated carbon. The analysis of the TGA and DTGA curves shows a slight initial mass loss (around 100 °C) may be associated with the removal of residual moisture or low-molecular-weight volatile compounds.^47,48^ After this stage, the material remains thermally stable, showing minimal mass variation up to approximately 550 °C, consistent with the thermal stability generally reported for activated carbons.^49,50^ This stabilization is also due to the high temperature and longer residence time (800 °C and 2 h) of pyrolysis, which promotes greater release of volatile compounds, leaving the activated carbon with low levels of volatile materials.^51^ The second peak (550–800 °C) corresponds to the degradation of oxygen-containing surface functional groups and the onset of deeper structural degradation.^52^

**Figure 5:**
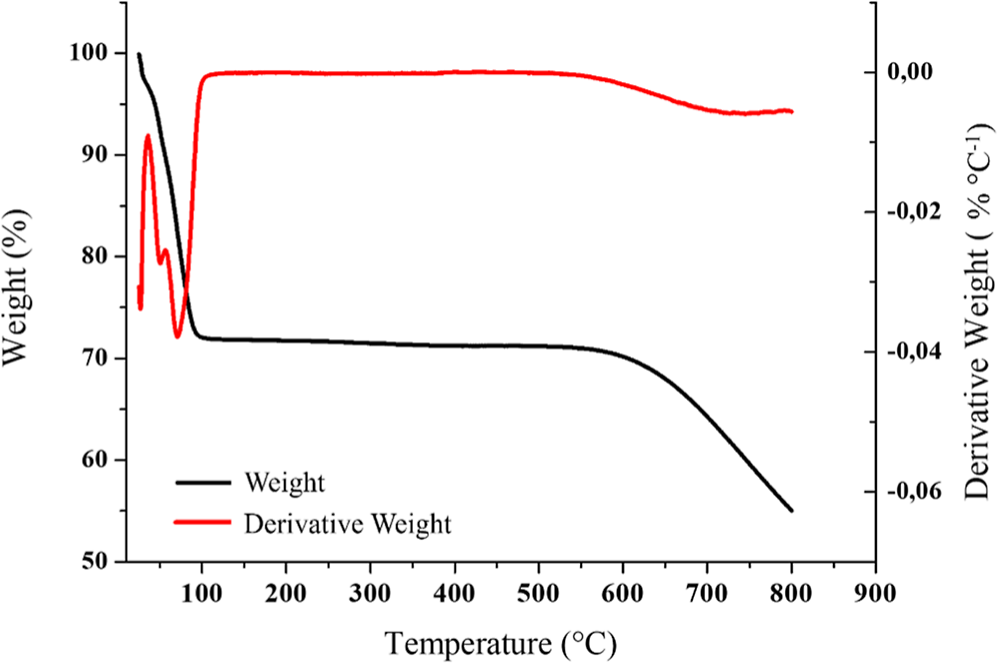
TGA and DTGA thermograms of activated carbon derived from grape pomace.

The results presented by Da Silva et al.^37^ had a different behavior: the grape pomace biochar presented great mass loss between 200 and 500 °C. The comparison of the results of this study with the ones obtained in the present work indicated that the carbonaceous matrix is stable probably due to the activation step that eliminated impurities. The absence of abrupt decomposition also suggests that the material maintains its structural integrity during thermal cycles, favoring its reusability.

#### 3.2.5 Differential Scanning Calorimetry

Differential scanning calorimetry (DSC) analysis of activated carbon derived from grape pomace was conducted between 25 and 300 °C under a nitrogen atmosphere (Figure 6). A broad endothermic peak between 45 and 110 °C corresponds to the release of adsorbed moisture, reflecting the hydrophilic nature of surface functional groups. No melting transition was observed in the DSC profile, which is consistent with the predominantly amorphous carbon structure. The absence of additional thermal events up to 300 °C suggests good thermal stability of the material within this temperature range.

**Figure 6:**
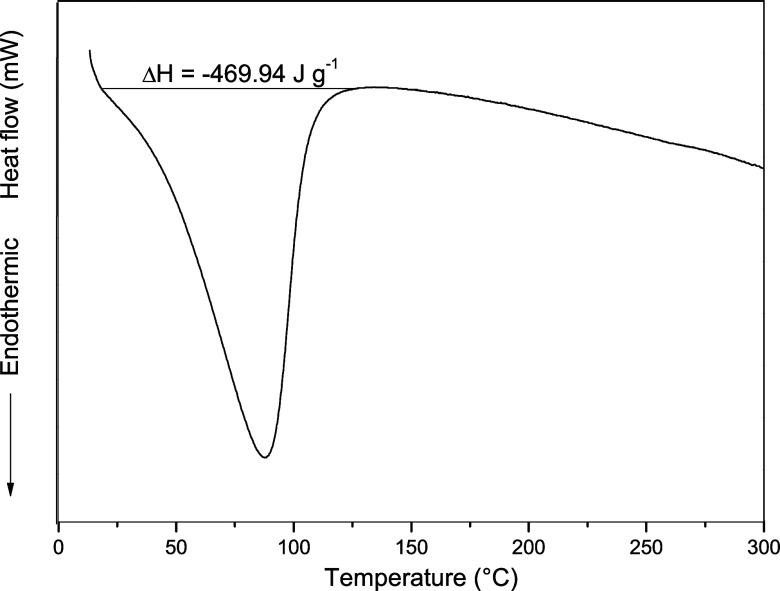
DSC thermogram of activated carbon derived from grape pomace recorded under a nitrogen atmosphere.

### 3.3 Kinetic Study

Figure 7 presents the adsorption kinetics of phenol at different initial concentrations (50–150 ppm). Experimental data are shown as points, while the lines represent the fitted kinetic models, which are analyzed and discussed later. The results showed that the higher the initial concentration of the solution (mg L^–1^), the greater the final absorption capacity value: the maximum absorption capacity reached approximately 113.12 mg g^–1^ for 150 mg L^–1^, 54.80 mg g^–1^ for 100 mg L^–1^, and 37.37 mg g^–1^ for 50 mg L^–1^. This occurs because at higher concentrations, more molecules are available to be adsorbed, resulting in greater saturation of the adsorbent. The behavior of the curves for all concentrations shows a rapid increase in q_t_ at the beginning of the adsorption process, followed by a plateau,^53^ indicating that equilibrium is reached at approximately 60 min under all conditions studied. This initial rapid adsorption is attributed to the high availability of active sites on the surface of the adsorbent. As these sites become occupied over time, the adsorption rate decreases until equilibrium is achieved, with no further significant uptake of phenol.

**Figure 7:**
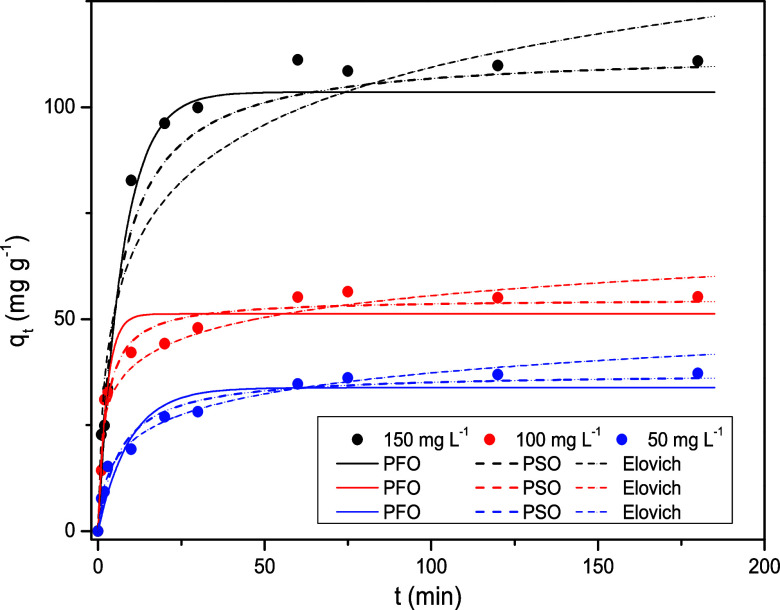
Adsorption kinetics of phenol using activated carbon derived from grape pomace.

The experimental data were fitted to the pseudo-first-order (PFO), pseudo-second-order (PSO), and Elovich models to describe the adsorption kinetics. The resulting kinetic parameters are summarized in Table 5, and the corresponding kinetic curves are presented in Figure 7. Overall, considering the high values of the coefficient of determination and the low values of the mean relative error presented in Table 5, the pseudo-second-order model provided the best fit for phenol concentrations of 100 and 150 mg L^–1^, indicating that the adsorption process is mainly governed by chemisorption mechanisms. However, at the lowest concentration (50 mg L^–1^), the Elovich model exhibited a slightly better correlation with the experimental data, suggesting that heterogeneous surface characteristics may play a more significant role under these conditions. This overall behavior is in agreement with several previous studies, in which the same model was also identified as the most appropriate for describing phenol adsorption on activated carbon.^54–57^ This behavior indicates that phenol adsorption on activated carbon occurs predominantly by chemisorption, involving interactions such as π–π conjugation and/or hydrogen bond formation.^58^

**Table 5:** Kinetic Model Parameters for Phenol Adsorption Using Activated Carbon Derived from Grape Pomace

		phenol concentration (mg L^–1^)^a^		
model		150	100	50
PFO	*k*_1_ (min^–1^)	0.13 ± 0.01^b^	0.38 ± 0.10^a^	0.10 ± 0.02^b^
	*q*_1_ (mg g^–1^)	103.56 ± 1.85^a^	51.23 ± 2.02^b^	33.84 ± 1.63^c^
	*R* ^2^	0.991	0.936	0.928
	χ^2^	7.39	4.59	12.61
	ARE (%)	6.15	9.82	17.45
PSO	*k*_2_ (g mg^–1^ min^–1^)	0.0015 ± 0.0002^c^	0.0079 ± 0.0018^a^	0.0041 ± 0.0008^b^
	*q*_2_ (mg g^–1^)	113.12 ± 2.34^a^	54.80 ± 1.65^b^	37.37 ± 1.31^c^
	*R* ^2^	0.991	0.973	0.970
	χ^2^	5.41	2.62	3.44
	ARE (%)	7.18	7.12	9.89
Elovich	*a* (g mg^–1^)	0.05 ± 0.01^c^	0.13 ± 0.08^a^	0.14 ± 0.02^a^
	*b* (mg g^–1^ min^–1^)	52.00 ± 38.46^b^	132.75 ± 39.38^a^	12.74 ± 6.45^c^
	*R* ^2^	0.942	0.962	0.989
	χ^2^	17.04	4.29	1.44
	ARE (%)	13.92	9.76	7.14

a Different letters within the same row indicate statistically significant differences (*p* < 0.05).

The adsorption equilibrium was reached within approximately 60 min for all concentrations with *k*_2_ values ranging from 0.0015 to 0.0079 g mg^–1^ min^–1^. Although these values are lower than those reported for *Cassia fistula* (0.0399–0.0738 g mg^–1^ min^–1^)^50^ and rice stalk–derived activated carbon (0.01 g mg^–1^ min^–1^),^59^ they are comparable to coconut shell (0.0002–0.0008 g mg^–1^ min^–1^).^60^ These discrepancies can be attributed to differences in the surface area, pore structure, and activation methods among the adsorbents.

### 3.4 Adsorption Isotherms

The phenol adsorption equilibrium curves, obtained at temperatures of 25, 45, and 55 °C for initial concentrations ranging from 50 to 500 mg L^–1^, are shown in Figure 8. Experimental data are shown as points, while the lines represent the fitted equilibrium models, which are analyzed and discussed later. It can be seen that the adsorption capacity increased progressively with rising temperature, reaching a maximum value of approximately 180 mg g^–1^ at 55 °C. This behavior indicates that the phenol adsorption process in activated carbon from grape pomace is endothermic, a conclusion that will be further confirmed by thermodynamic parameters.

**Figure 8:**
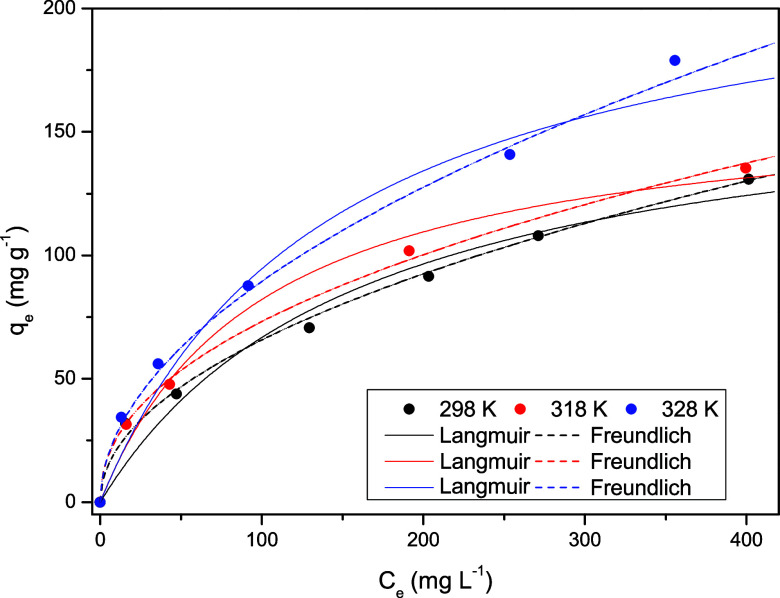
Adsorption isotherms of phenol using activated carbon derived from grape pomace.

The Langmuir and Freundlich models were applied to interpret the adsorption equilibrium of phenol on activated carbon from grape pomace, and the results are presented in Table 6. Both models were found to fit the experimental phenol adsorption data well. However, the Freundlich model proved to be more suitable as it resulted in higher *R* ^2^ values and lower χ^2^ and ARE values. Thus, this model was selected to represent the adsorption equilibrium. In addition, the fit by the Freundlich model indicates that the process occurs in multilayers on heterogeneous surfaces, in which the adsorption heat and surface affinities diverge unevenly. This finding is in agreement with previous studies, which also reported that the Freundlich model more appropriately describes the adsorption of phenol on activated carbon.^54,61,62^

**Table 6:** Isotherms Model Parameters for Phenol Adsorption Using Activated Carbon Derived from Grape Pomace

		*T* (°C)^a^		
isothermal model	parameters	25	45	55
Langmuir	*q*_m_ (mg g^–1^)	174.35 ± 32.44^b^	164.24 ± 14.55^b^	231.15 ± 32.04^a^
	*K*_L_ (L mg^–1^)	0.0062 ± 0.0028^b^	0.0100 ± 0.0020^a^	0.0069 ± 0.0020^b^
	*R* ^2^	0.943	0.981	0.941
	χ^2^	18.93	3.80	11.80
	ARE (%)	13.88	9.90	15.10
Freundlich	*K*_F_ ((mg g^–1^) (mg L^–1^)^−1/*n*^)	6.80 ± 2.25^a^	9.02 ± 1.50^a^	8.43 ± 2.48^a^
	*n*	2.03 ± 0.25^a^	2.20 ± 0.14^a^	1.95 ± 0.20^a^
	*R* ^2^	0.993	0.996	0.988
	χ^2^	1.38	0.25	0.46
	ARE (%)	4.80	2.68	5.07

a Different letters within the same row indicate statistically significant differences (*p* < 0.05).

To contextualize the performance of the grape pomace-derived activated carbon, Table 7 compares its maximum phenol adsorption capacity with those of other adsorbents reported in the literature. The obtained value (*q*_m_ = 231 mg g^–1^ at 55 °C) is higher than or comparable to those of other biomass-based activated carbons, indicating the potential of grape pomace as a low-cost precursor for efficient phenol removal. Although some studies report higher adsorption capacities, the performance of the material presented here remains competitive within the range of activated carbons derived from biomass. Moreover, the use of an abundant agro-industrial residue enhances the material’s sustainability and practical applicability. The relatively high adsorption capacities achieved can be attributed to the combination of surface functional groups’ (−OH, C=O, C–O), micro- and mesoporosity, and high surface area identified in Sections 3.2.1–3.2.3.

**Table 7:** Comparison of Phenol Adsorption Capacities Reported in the Literature for Activated Carbon Derived from Agro-industrial Residues

adsorbent	*q*_m_ (mg g^–1^)	reference
*Moringa oleifera*, sesame, and baobab husks activated carbon	717	^63^
babassu activated carbon	597	^64^
food waste activated carbon	568	^65^
oak wood charcoal activated carbon	250	^66^
grape pomace activated carbon (this study)	231	-
coconut shell powdered activated carbons	213	^60^
rice husk activated carbon	201	^67^
coal activated carbon	170	^60^
rattan sawdust activated carbon	149	^68^
olive pits activated carbon	120	^61^
rice stalk activated carbon	40	^59^
bagasse fly ash activated carbon	26	^69^
*Borassus flabellifer* fruit husk activated carbon	17	^70^

### 3.5 Thermodynamic Parameters

The thermodynamic performance of phenol adsorption on activated carbon obtained from grape pomace was evaluated based on the variation in Gibbs free energy (Δ*G*°), enthalpy (Δ*H*°), and entropy (Δ*S*°), as shown in Table 8. The negative values of Δ*G*° indicated that adsorption occurred spontaneously and favorably with the most negative value observed at 55 °C, which shows that increasing the temperature favors the process. The positive value of Δ*H* confirms the endothermic nature of adsorption, a result that can be attributed to the desolvation of water molecules present on the surface of the adsorbent, a necessary step for the subsequent adsorption of phenol molecules. Therefore, the Δ*H*° value suggests physisorption of phenol on activated carbon from grape pomace. In addition, the positive Δ*S*° value shows that the disorder at the solid–liquid interface increased after adsorption.^71^ It was also observed that only the entropy variation contributed to obtaining negative Δ*G*° values. This shows that the adsorption of phenol on activated carbon from grape pomace was an entropy-controlled phenomenon. Similar thermodynamic behavior was found by Almahbashi et al.^59^ in the adsorption of phenol on activated carbon derived from rice straw.

**Table 8:** Thermodynamic Parameters for Phenol Adsorption by Activated Carbon from Grape Pomace

*T* (°C)	Δ*G*° (kJ mol^–1^)	Δ*H*° (kJ mol^–1^)	Δ*S*° (J mol^–1^ K^–1^)
25	–18.85	6.2	83.80
45	–20.03		
55	–21.53		

The apparent discrepancy between the PSO kinetic fit, which implies chemisorption, and the low enthalpy change (Δ*H*°), indicative of physisorption, suggests that phenol adsorption on ZnCl_2_-activated grape pomace carbon is predominantly governed by physisorption mechanisms. These include π–π interactions between the aromatic rings of phenol and graphitic domains as well as hydrogen bonding with oxygenated surface functional groups. The PSO kinetic behavior reflects the rate-limiting role of adsorption onto specific active sites, particularly at higher solute concentrations (100 and 150 mg L^–1^). Therefore, phenol removal probably occurs predominantly via physisorption (π–π and H-bonding) on a heterogeneous carbon surface, with a possible contribution of stronger site-specific interactions that become more noticeable at higher initial concentrations.

### 3.6 Adsorption–Desorption Cycles

Desorption analysis provides insights into the potential reuse of activated carbon derived from grape pomace. The adsorption–desorption cycles were evaluated using NaOH as the regenerating agent. Figure 9 presents the desorption percentages of phenol over five consecutive cycles. As shown in Figure 9, NaOH enabled the reuse of the activated carbon for up to five cycles while maintaining both the satisfactory removal efficiency and physical integrity of the adsorbent. The average desorption efficiencies obtained were 48% in the first cycle, 37% in the second, 43% in the third, 44% in the fourth, and 43% in the fifth cycle.

**Figure 9:**
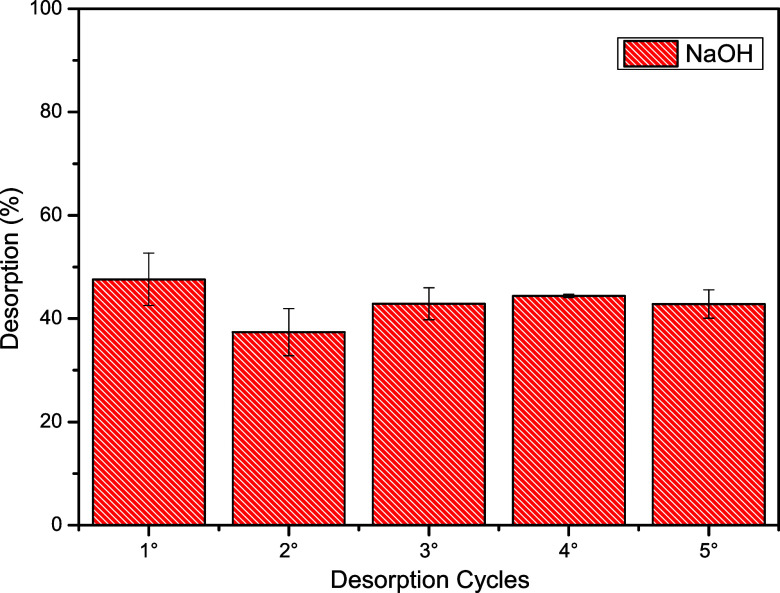
Adsorption–desorption cycles of phenol by activated carbon produced from grape pomace.

The regeneration capacity of NaOH can be attributed to its reaction with phenol, forming the soluble salt C_6_H_5_O–Na^+^, which facilitates the desorption of phenol from the activated carbon surface.^72^ Moreover, the adsorption of phenol molecules onto activated carbon is likely governed by multiple interaction mechanisms, including electrostatic forces, π–π interactions, electron donor–acceptor interactions, and hydrogen bonding.^73^

Similar regeneration efficiencies have been reported for other phenol adsorbents, such as activated carbons from coconut shell (40–50%)^60^ black wattle bark waste (30–70%),^71^ *C. fistula* (75–80%).^50^ These results suggest that the strong interaction between phenol molecules and surface functional groups limits complete desorption, although the material maintains a structural stability and reasonable reusability. To improve reusability, alternative strategies could be considered, such as the use of mild acids or organic solvents for desorption, thermal regeneration, or combined methods, which may enhance the desorption efficiency while reducing the environmental impact.

## 4 Safe Disposal of the Spent Adsorbent

After the adsorption experiments, spent activated carbon was filtered, dried, and properly disposed of as hazardous solid waste for subsequent treatment.

## 5 Conclusion

Activated carbon produced from grape pomace (*V. labrusca*, Bordeaux variety) using ZnCl_2_ chemical activation and optimized carbonization conditions demonstrated favorable physicochemical properties for adsorption applications. Factorial design revealed that the carbonization temperature significantly influenced phenol adsorption capacity, with higher temperatures improving performance. The selected condition (800 °C, 120 min) resulted in high surface area, micro- and mesoporosity, and the presence of functional groups that favor adsorption. Characterization analyses confirmed the porosity developed by the material and its thermal stability. Adsorption kinetic experiments showed that the activated carbon efficiently removed phenol from aqueous solutions, reaching a capacity of up to 113.12 mg g^–1^ depending on the initial concentration. Kinetic modeling indicated that pseudo-second-order phenol adsorption behavior was best suited regardless of the initial concentration. Regarding the equilibrium studies, the maximum adsorption capacity was 180 mg g^–1^. Isotherm modeling indicated that the Freundlich model was the most appropriate. Thermodynamic parameters indicated that the higher the temperature of the aqueous system, the greater the adsorption capacity. Reuse indicated that after five adsorption–desorption cycles, the adsorbent demonstrated mechanical stability, maintaining its physical integrity. Overall, this work highlights the potential of utilizing agro-industrial waste for the development of effective and sustainable adsorbents for environmental remediation.
